# Selective abdominal venous congestion induces adverse renal and hepatic morphological and functional alterations despite a preserved cardiac function

**DOI:** 10.1038/s41598-018-36189-3

**Published:** 2018-12-10

**Authors:** Jirka Cops, Wilfried Mullens, Frederik H. Verbrugge, Quirine Swennen, Bart De Moor, Carmen Reynders, Joris Penders, Ruth Achten, Ann Driessen, Amélie Dendooven, Jean-Michel Rigo, Dominique Hansen

**Affiliations:** 1 BIOMED – Biomedical Research Institute, Faculty of Medicine and Life Sciences, Hasselt University, Diepenbeek, Belgium; 2 Doctoral school for Medicine and Life Sciences, Hasselt University, Diepenbeek, Belgium; 3 Department of Cardiology, Ziekenhuis Oost-limburg, Genk, Belgium; 4 Department of Nephrology, Jessa Ziekenhuis, Hasselt, Belgium; 5 Clinical laboratory, Ziekenhuis Oost-Limburg, Genk, Belgium; 6 Department of Pathology, Jessa Ziekenhuis, Hasselt, Belgium; 7 Department of Pathology, Universitair Ziekenhuis Antwerpen, University of Antwerp, Edegem, Belgium; 8 REVAL – Rehabilitation Research Center, Faculty of Rehabilitation Sciences, Hasselt University, Diepenbeek, Belgium; 9 Heart Centre Hasselt, Jessa Hospital, Hasselt, Belgium

## Abstract

Venous congestion is an important contributor to worsening renal function in heart failure and the cardiorenal syndrome. In patients, it is difficult to study the effects of isolated venous congestion on organ function. In this study, the consequences of isolated abdominal venous congestion on morphology and function of the kidneys, liver and heart were studied in a rat model. Twelve sham-operated (SHAM) male Sprague Dawley rats were compared to eleven inferior vena cava-constricted (IVCc) rats for twenty-one weeks. Abdominal venous pressure was significantly higher in the IVCc versus SHAM group (p < 0.0001). Indices of liver and kidney weight, function and morphology, inflammation as well as collagen deposition were significantly increased in the IVCc compared to SHAM group, (p < 0.05). Echocardiographic and hemodynamic parameters were largely unaffected by abdominal venous congestion. In this rat model of isolated abdominal venous congestion, retrogradely conducted glomerular hypertension without a concomitant change in glomerular filtration rate was observed. Adverse short-term hepatic morphological alterations were developed which explain the observed organ function dysfunction. Importantly, cardiac function remained comparable between both groups. This study provides relevant insight in the pathophysiology of abdominal congestion on organ function.

## Introduction

Heart failure is defined as a condition whereby the heart is not able to maintain adequate organ perfusion in the face of normal filling pressures. It comprises both forward failure (i.e., impaired cardiac output) and backward failure (i.e., venous congestion). The latter is an established mechanism driving disease progression^1–3^.

Venous congestion is caused by overfilling of the central venous capacitance veins^4^ as a result of the failing heart with unrestrained sodium and water retention and neurohumoral activation, which may ultimately lead to organ injury and failure when severe and long-standing. Different target organs such as the heart, kidneys, liver, intestines and lungs are potentially implied. Pulmonary congestion mainly develops in case of increased left ventricular filling pressures, whereas abdominal congestion is a classic sign of right-sided heart failure^5^. It has been shown that both central venous pressure (CVP) and intra-abdominal pressure, as measures for venous congestion, are associated with kidney dysfunction in heart failure^1,6–9^ as well as in other settings^10–12^.

It remains unclear how isolated venous congestion, separate from cardiac dysfunction, leads to heart and kidney failure in patients^13,14^. However, this can be studied in a rat model with selectively increased abdominal venous pressure, which can be accomplished by permanently constricting the thoracic inferior vena cava (IVC)^15^. It has already been shown that such a model did not compromise cardiac preload or contractile function, while important alterations in kidney morphology and function were observed after twelve weeks^15^. However, hepatic function may also be affected by abdominal venous congestion. Accordingly, this study has been prolonged to an observational period of twenty-one weeks in which cardiac, renal and by extension hepatic function and morphology are examined. Furthermore, mechanisms responsible for organ dysfunction, are investigated in depth.

Therefore, the objective of this study is to identify the deleterious effects of abdominal venous congestion on hepatic, renal and cardiac morphology and function as well as to investigate the underlying structural alterations that might explain these effects. The unique advantage of our rat model is that the effects of backward failure on congestion-related diseases can be investigated separately from forward failure.

## Results

### Surgical constriction of the IVC induces abdominal venous congestion

Baseline pre-surgical physical, blood, urinary and echocardiographic parameters were not statistically different between IVCc and SHAM rats, except for a significantly lower body weight and urine volume (p = 0.04) and a significantly higher heart rate (p = 0.01) in IVCc versus sham-operated rats (Tables 1–2).

**Table 1:** Baseline blood and urinary parameters and after twenty-one weeks of follow-up.

	Baseline (week 0)			Week 21		
	SHAM	IVCc	p-value	SHAM	IVCc	p-value
Body weight (g)	252 [180;257]	175 [161;234]*	0.04	690 [621;725]	641 [579;698]	0.22
Plasma creatinine (mg/dl)^#^	0.18 [0.15;0.23]	0.14 [0.10;0.21]	0.20	0.26 [0.23;0.27]	0.29 [0.28;0.42]**	0.001
Plasma cystatin C (mg/dl)^#^	2.19 [1.12:2.49]	1.58 [1.33;2.13]	0.76	1.60 [0.95;1.85]	2.10 [1.60;2.25]	0.26
Urinary albumin (mg/g crea)^#^	94.9 [72.4;171.5]	113.9 [82.5;197.6]	0.49	129.8 [56.5;247.0]	87.8 [65.9;195.1]	0.74
Urinary KIM-1 (ng/g crea)	1991 [1538;2886]	2149 [1524;2684]	0.65	544 [449;639]	588 [390;680]	0.82
Urinary creatinine excretion (mg/24 h)^#^	51.40 [36.85;58.78]	55.80 [48.10;65.50]	0.36	51.40 [36.85;58.78]	55.80 [48.10;65.50]	0.39
Creatinine clearance (ml/min/kg)	5.91 [3.90;6.45]	5.75 [4.01;7.65]	0.70	8.40 [5.93;9.62]	8.61 [5.91;9.36]	0.77
Plasma urea (mg/dl)^#^	25 [18;30]	22 [19;25]	0.41	29 [27;33]	33 [29;37]	0.16
Urinary urea excretion (mg/24 h)	4259 [2553;5085]	4474 [2570;5340]	0.39	4942 [3845;6536]	5349 [3783;7669]	0.58
Urea clearance (ml/min/kg)^#^	2.898 [2.470;3.373]	3.236 [2.425;3.902]	0.63	0.007 [0.004;0.008]	0.006 [0.003;0.007]	0.95
Water intake (ml/24 h)^#^	30 [30;35]	25 [23;30]	0.11	38 [30;51]	40 [30;45]	0.73
Urine volume (ml/24 h)	6 [5;11]	5 [4;6]*	0.04	19 [16;24]	17 [14;22]	0.62
Plasma bilirubin (mg/dl)	0.03 [0.02;0.04]	0.04 [0.03;0.05]	0.07	0.04 [0.03;0.05]	0.07 [0.06;0.08]****	<0.0001
Plasma CRP (mg/dl)^#^				0.02 [0.0;0.04]	0.23 [0.08;0.34]*	0.019
Plasma aldosterone (ng/l)				175.9 [137.7;298.7]	262.2 [136.3;322.4]	0.48

Data are shown as median [25^th^ percentile;75^th^ percentile] in sham-operated (SHAM, n = 12) and IVC-constricted rats (IVCc, n = 11). Baseline plasma CRP and aldosterone levels are not available due to a too small sample volume. Data were analyzed using an unpaired t-test when parametrically distributed according to the Shapiro-Wilk normality test. ^#^ denotes non-parametrically distributed data, which were analyzed using a Mann-Whitney test. * denotes p < 0.05, ** denotes p < 0.01, **** denotes p < 0.0001. The observed statistical power of inter-group comparisons is 0.42 and 0.59 for baseline body weight and urine volume respectively and 0.70, 0.99 and 0.58 for plasma creatinine, bilirubin and CRP after 21 weeks, respectively. KIM-1 = kidney injury molecule 1, CRP = C-reactive protein, IVC = inferior vena cava, IVCc = IVC-constricted rats.

**Table 2:** Baseline conventional echocardiography parameters and after twenty-one weeks of follow-up.

	Baseline (week 0)			Week 21		
	SHAM	IVCc	p-value	SHAM	IVCc	p-value
LVEDD (mm)	5.3 [4.9;5.8]	5.0 [4.8;5.1]	0.18	7.8 [7.4;8.4]	7.7 [6.7;8.0]	0.33
LVESD (mm)	2.7 [2.3;3.1]	2.9 [2.5;3.2]	0.29	4.3 [3.8;4.7]	3.9 [3.0;4.4]	0.14
PWT (mm)	0.97 [0.82;1.02]	0.85 [0.74;0.94]	0.16	0.78 [0.72;0.86]	0.92 [0.83;1.01]*	0.04
AWT (mm)^#^	0.76 [0.63;0.87]	0.66 [0.57;0.66]	0.05	0.73 [0.70;0.94]	0.80 [0.70;0.92]	0.63
HR (bpm)^#^	398 [388;430]	441 [416;455]**	0.006	338 [318;356]	346 [334;356]	0.22
EDV (µl)	148 [124;177]	135 [105;148]	0.26	471 [404;577]	403 [323;482]	0.31
ESV (µl) ^#^	8 [6;13]	11 [8;13]	0.42	32 [22;40]	19 [15;33]	0.13
CO (ml/min)^#^	56 [46;64]	55 [46;61]	0.62	158 [125;179]	132 [105;157]	0.22

Data are shown as median [25^th^ percentile;75^th^ percentile] in sham-operated (SHAM, n = 12) and IVC-constricted rats (IVCc, n = 11). Data were analyzed using an unpaired t-test when parametrically distributed according to the Shapiro-Wilk normality test. ^#^ denotes non-parametrically distributed data, which were analyzed using a Mann-Whitney test. * denotes p < 0.05, ** denotes p < 0.01. The observed statistical power of inter-group comparisons is 0.83 for baseline HR and 0.59 for PWT after 21 weeks. LVEDD = left ventricular end-diastolic diameter, LVESD = left-ventricular end-systolic diameter, PWT = posterior wall thickness, AWT = anterior wall thickness, HR = heart rate, EDV = end diastolic volume, ESV = end systolic volume, CO = cardiac output, IVC = inferior vena cava, IVCc = IVC-constricted rats.

**Figure 1:**
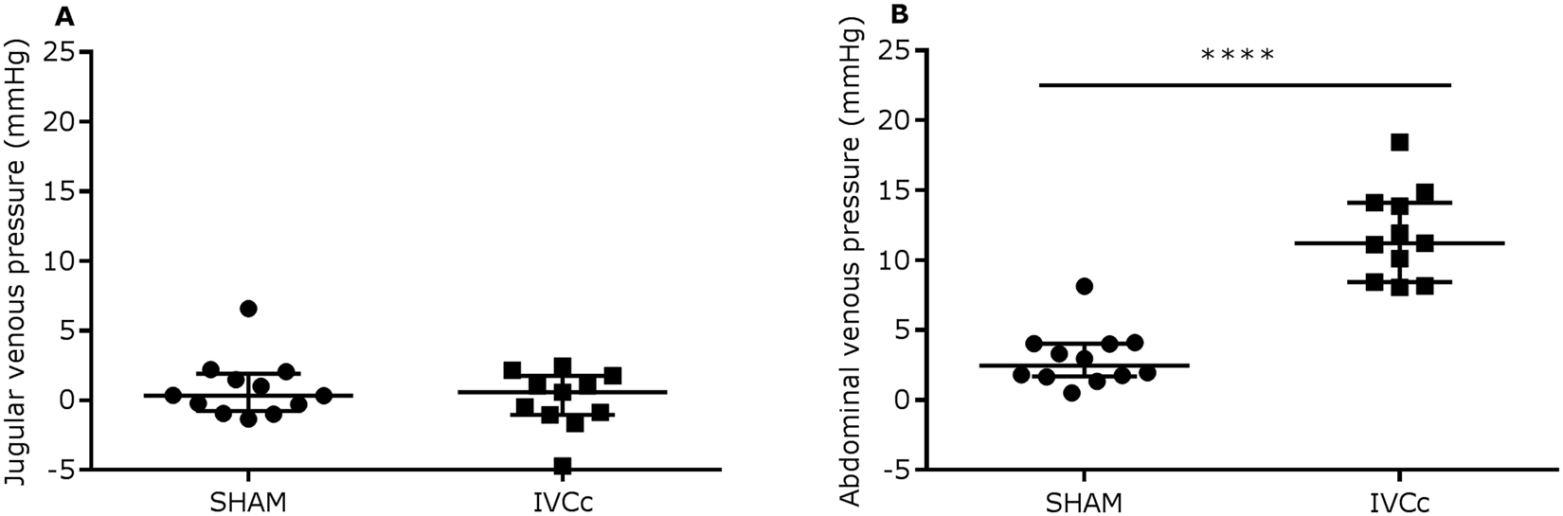
Permanent inferior vena cava constriction is sufficient to increase the abdominal venous pressure. (**A**) jugular venous pressure above the constriction level and (**B**) abdominal venous pressure below the constriction level of sham-operated (SHAM, n = 12) and IVC-constricted rats (IVCc, n = 11). Data were analyzed using a Mann-Whitney test (**A**) or an unpaired t-test (**B**), according to the Shapiro-Wilk normality test. Data are shown as median, 25^th^ percentile, 75^th^ percentile, minimum and maximum. **** denotes p < 0.0001. The observed statistical power of inter-group comparisons is 1 in (**B**). IVC = inferior vena cava, IVCc = IVC-constricted rats.

At 21 weeks after surgery, jugular venous pressure was not statistically different between both groups (0.4 [−0.8;1.9] mmHg in the SHAM group versus 0.6 [−1.0;1.8] mmHg in the IVCc group, p = 0.74 (median [25^th^;75^th^ percentile]); Fig. 1A). Contrarily, abdominal venous pressure increased significantly in IVCc compared to SHAM rats (11.2 [8.5;14.1] mmHg versus 2.5 [1.7;4.0] mmHg, respectively; p < 0.0001; Fig. 1B). Physical parameters of IVCc versus sham-operated rats of both cohorts are shown in Table 3. After 21 weeks of abdominal venous congestion, liver weight/tibia length ratio and spleen weight/tibia length ratio were significantly greater in the IVCc group (Table 3, p < 0.05), heart weight/tibia length ratio was significantly smaller in the IVCc group (Table 3, p < 0.01) and no significant differences in body weight (Table 1), body weight gain and kidney weight/tibia length ratio were observed between groups (Table 3). Plasma CRP levels were significantly higher in IVCc-rats (p < 0.05, Table 1).

**Table 3:** Physical and cardiac hemodynamic parameters after twenty-one week of follow-up.

Week 21	SHAM	IVCc	p-value
Body weight gain (g/weeks)	461 [430;489]	462 [403;502]	0.88
Liver weight/tibia length (mg/mm)	431.8 [400.1;469.6]	498.4 [456.4;522.0]*	0.03
Spleen weight/tibia length (mg/mm)^#^	19.4 [16.4;22.4]	25.2 [19.1;26.5]*	0.04
Heart weight/tibia length (mg/mm)	43.8 [40.9;47.0]	35.5 [33.7;41.5]**	0.007
Kidney weight/tibia length (mg/mm)^#^	86.9 [83.3;95.9]	80.1 [72.7;88.1]	0.21
LVP (mmHg)^#^	105.3 [92.2;110.2]	101.9 [93.4;106.2]	0.58
LVEDP (mmHg)	16.9 [80;21.8]	13.7 [10.6;20.1]	0.91
dP/dt_max_ (mmHg/s)	2049 [1860;2591]	[2027;3300]	0.20
dP/dt_min_ (mmHg/s)	−1786 [−1991;−1485]	−2096 [−2929;−1489]	0.20
Tau (s)^#^	0.016 [0.007;0.112]	0.010 [0.009;0.380]	0.59

Data are shown as median [25^th^ percentile;75^th^ percentile] in sham-operated (SHAM, n = 12) and IVC-constricted rats (IVCc, n = 11). Data were analyzed using an unpaired t-test when parametrically distributed according to the Shapiro-Wilk normality test. ^#^ denotes non-parametrically distributed data, which were analyzed using a Mann-Whitney test. * denotes p < 0.05, ** denotes p < 0.01. The observed statistical power of inter-group comparisons is 0.60, 0.55 and 0.83 for liver weight/tibia length ratio, spleen/weight tibia length ratio and heart weight/tibia length ratio, respectively. LVP = left ventricular pressure, LVEDP = left ventricular end-diastolic pressure, dP/dt_max_ = maximum value of the first derivate of LV pressure, dP/dt_min_ = minimum value of the first derivate of LV pressure, tau = time constant of LV pressure decay during the isovolumic relaxation period, IVC = inferior vena cava, IVCc = IVC-constricted rats.

### Abdominal venous congestion increases the glomerular surface area and width of Bowman’s space without major impact on the glomerular filtration rate

**Figure 2:**
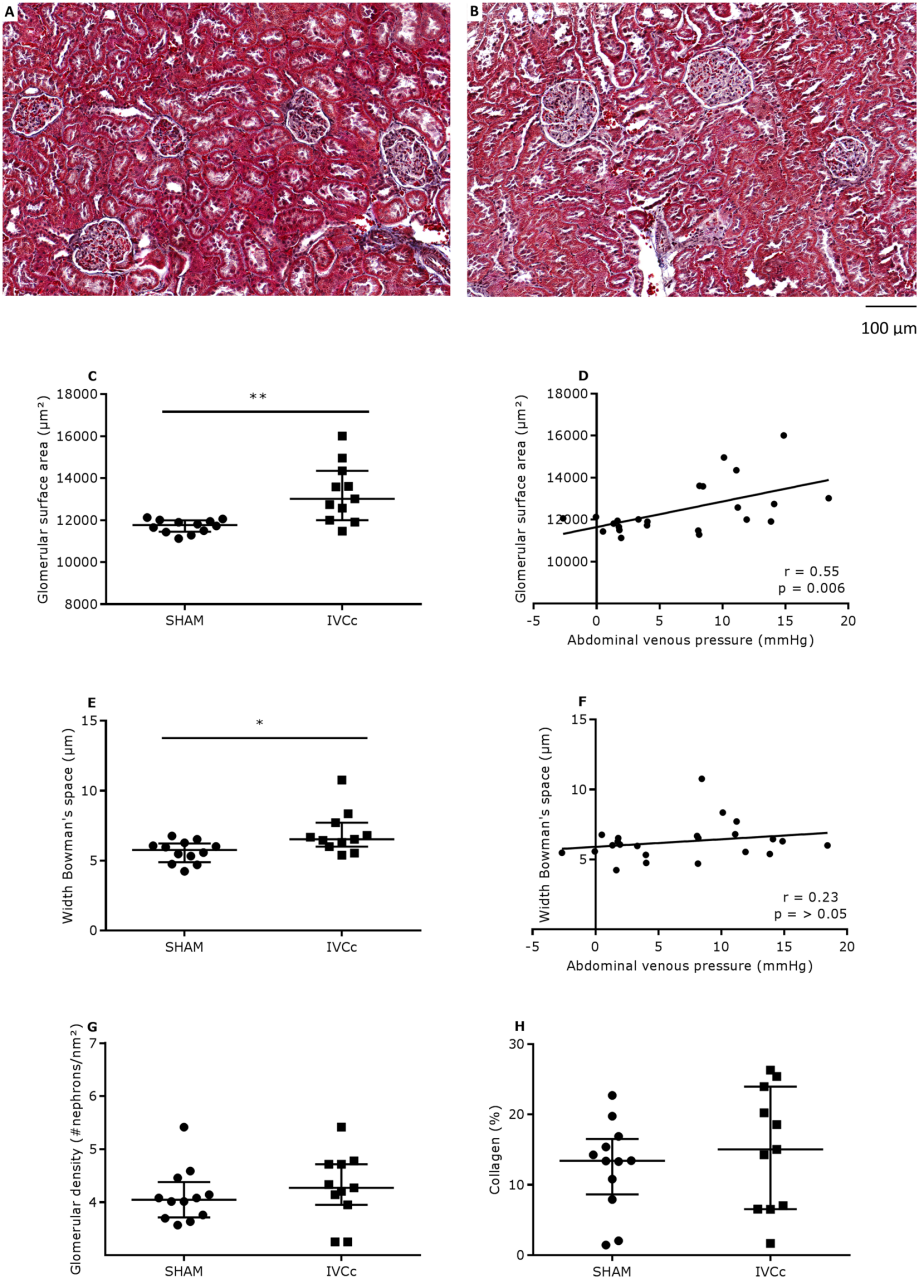
Abdominal venous congestion alters kidney morphology. Representative Masson trichrome staining in transverse sections of a kidney of (**A**) a sham-operated and (**B**) an IVC-constricted rat, twenty-one weeks after surgery. Magnification is 20×, scale bar is 100 µm. (**C**) Glomerular surface area, (**D**) glomerular surface area expressed as a function of abdominal venous pressure, (**E**) width of Bowman’s space, (**F**) width of Bowman’s space expressed as a function of abdominal venous pressure, (**G**) glomerular density and (**H**) quantification of renal collagen of sham-operated (SHAM, n = 12) and IVC-constricted rats (IVCc, n = 11). Data were analyzed using an unpaired t-test (**C**,**G**) or a Mann-Whitney test (**E**,**H**) and relations were examined by Pearson (**D**) or Spearman correlations (**F**), according to the Shapiro-Wilk normality test. Data are shown as median, 25^th^ percentile, 75^th^ percentile, minimum and maximum. * denotes p < 0.05, ** denotes p < 0.01. The observed statistical power of inter-group comparisons is 0.94 in (**C**) and 0.70 in (**E**). IVC = inferior vena cava, IVCc = IVC-constricted rats.

**Figure 3:**
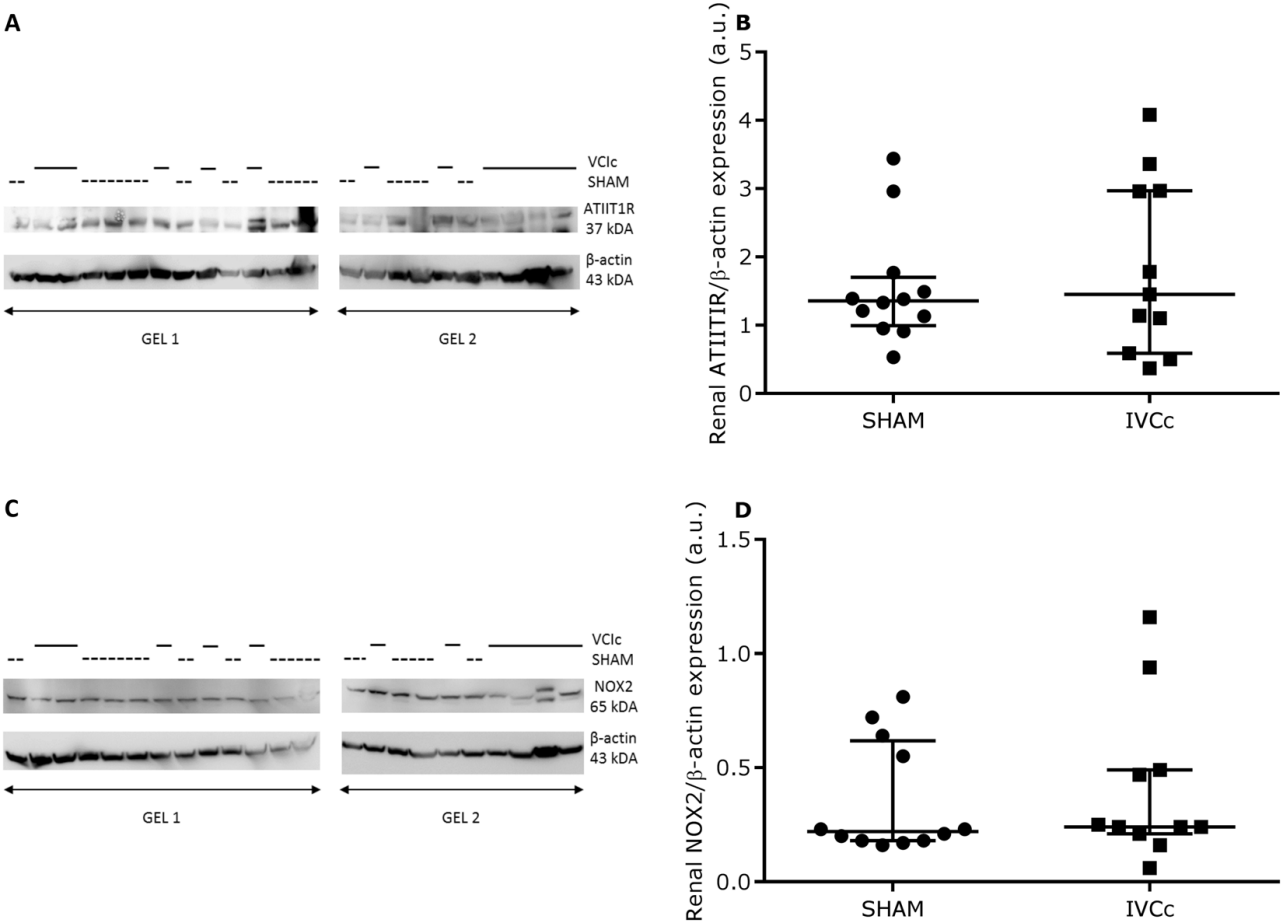
Abdominal venous congestion does not lead to renal RAAS system hyperactivation or oxidative stress. (**A**) Representative western blot for renal angiotensin II type I receptor (ATIIT1R) and β-actin of sham-operated (SHAM, n = 12) and IVC-constricted rats (IVCc, n = 11), twenty-one weeks after surgery. (**B**) Quantitative analysis of renal ATIIT1R protein expression normalized to β-actin of SHAM and IVCc rats. (**C**) Representative western blot for renal NAPDH oxidase 2 (NOX2) and β-actin of SHAM and IVCc rats. (**D**) Quantitative analysis of renal NOX2 protein expression normalized to β-actin of SHAM and IVCc rats. Samples for western blot for ATTIIT1R and NOX2 were derived from the same animal experiment. However, samples were dived over two gels, due to lack of space, and blots were processed in parallel. Solid line = IVC-constricted rats, dotted line = sham-operated rats. Data were analyzed using a Mann-Whitney test (**B**,**D**). Data are shown as median, 25^th^ percentile, 75^th^ percentile, minimum and maximum. IVC = inferior vena cava, IVCc = IVC-constricted rats, ATIIT1R = angiotensin II type I receptor, NOX2 = NAPDH oxidase 2.

Glomerular surface area and width of Bowman’s space were significantly greater in the IVCc versus SHAM group (p < 0.01 and p < 0.05, respectively; Fig. 2A–C,E). Twenty-one weeks after surgery, only the increase in glomerular surface area correlated significantly with the abdominal venous pressure (r = 0.55, p < 0.01; Fig. 2D). No correlation between the increase in width of Bowman’s space and the abdominal venous pressure was observed (r = 0.23, p = 0.30; Fig. 2F). Glomerular density and renal collagen deposition did not differ between both groups (Fig. 2G,H). With a similar water intake in both groups, 24 h urine volume was also comparable among IVCc versus sham-operated rats. Glomerular filtration rate, estimated by the renal creatinine clearance, did not differ between groups 21 weeks after surgery. However, plasma creatinine was significantly greater in the IVC-constricted versus sham-operated group (p < 0.01), while plasma cystatin C and urea and urinary KIM-1, albumin, creatinine excretion, urea excretion and urea clearance did not differ between both groups (Table 1). Plasma aldosterone (Table 1) and protein expression levels of renal angiotensin II type I receptor (ATIIT1R, Fig. 3A,B) and NAPDH oxidase 2 (NOX2, Fig. 3C,D) did not differ between both groups.

### IVC-constricted rats develop hepatic fibrosis

**Figure 4:**
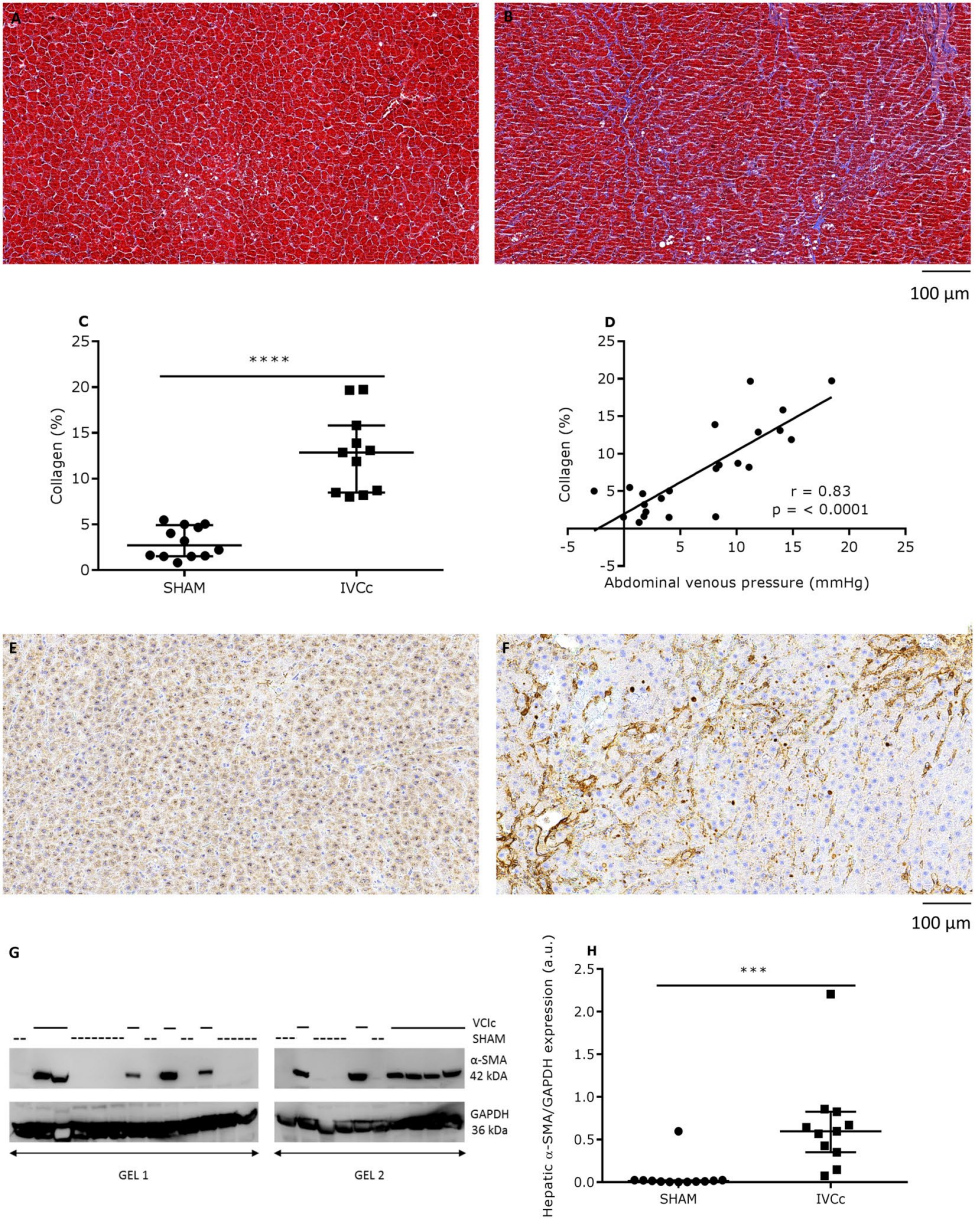
Abdominal venous congestion leads to hepatic fibrosis and increases hepatic α-SMA expression. Representative Masson trichrome staining in hepatic transverse sections of (**A**) a sham-operated (SHAM) and (**B**) an IVC-constricted rat (IVCC), twenty-one weeks after surgery. Magnification is 20×, scale bar is 100 µm. (**C**) Quantification of hepatic collagen and (**D**) total collagen expressed as a function of abdominal venous pressure of SHAM (n = 12) and IVCc rats (n = 11). Representative α-SMA immunohistochemical staining in hepatic transverse sections of (**E**) a SHAM rat and (**F**) an IVCc rat. Magnification is 20×, scale bar is 100 µm. (**G**) Representative western blot for hepatic α-SMA and GAPDH of SHAM and IVCc rats. Samples were derived from the same animal experiment. However, samples were dived over two gels, due to lack of space, and blots were processed in parallel. Solid line = IVC-constricted rats, dotted line = sham-operated rats. (**H**) Quantitative analysis of hepatic α-SMA protein expression normalized to GAPDH of SHAM and IVCc rats. Data were analyzed using an unpaired t-test (**C**) or a Mann-Whitney test (**H**), according to the Shapiro-Wilk normality test. Relations were examined by Pearson correlations (**D**). Data are shown as median, 25^th^ percentile, 75^th^ percentile, minimum and maximum. *** denotes p < 0.001, **** denotesp < 0.0001. The observed statistical power of inter-group comparisons is 0.99 in (**C**) and 0.91 in (**H**). IVC = inferior vena cava, IVCc = IVC-constricted rats, α-SMA = alpha-smooth muscle actin.

IVCc rats demonstrated significantly increased plasma bilirubin levels after 21 weeks (p < 0.0001, Table 1). Histological examination revealed that fibrosis was markedly augmented in the liver of IVCc rats (p < 0.0001; Fig. 4A–C). Furthermore, collagen deposition correlated significantly with the abdominal venous pressure (r = 0.83, p < 0.0001; Fig. 4D). As indicated by immunostaining, an increased number of α-SMA-positive myofibroblasts was observed in IVCc versus sham-operated rats (representative pictures: Fig. 4^E^,F). Fig. 4G is a representative example of a western blot for α-SMA and GAPDH from liver samples. As shown in Fig. 4H, protein expression levels of α-SMA were significantly increased in IVCc rats, 21 weeks after surgery (p < 0.001).

### Cardiac function remains unaltered

Echocardiographic parameters (Table 2), cardiac hemodynamic parameters (Table 3) and cardiac collagen deposition (Supplemental Fig. S1) were not statistically different between both groups after 21 weeks of surgery, except for a significantly increased posterior wall thickness in IVC-constricted rats (p < 0.05).

## Discussion

This study explored the effects of isolated abdominal venous congestion on heart, kidneys and liver morphology and function. This may offer insight into how backward failure affects liver and kidney function, independent of cardiac failure. Our main observations are: (1) surgical constriction of the thoracic IVC succeeds to increase the abdominal venous pressure significantly, associated with a systemic inflammatory status; (2) abdominal congestion is associated with increased glomerular surface area and Bowman’s width, suggesting the presence of glomerular hypertension; but (3) no significant change in glomerular filtration rate is observed; (4) abdominal venous congestion is associated with hepatic fibrosis and (5) cardiac function is not compromised by selective abdominal venous congestion.

### Thoracic IVC constriction is efficient to increase the abdominal venous pressure without compromising cardiac preload

A rat model has been developed with increased abdominal venous pressure but without major impact on cardiac output or systemic hemodynamics. In this model, the thoracic IVC is narrowed through a surgically placed ligature^15^. The average abdominal venous pressure in this model typically rises to 8–18 mmHg, which is sustained over time. In patients, an increased central venous pressure is defined as values of >8 mmHg^1^. In our model of abdominal hypertension, all abdominal organs are exposed to increased venous pressure and thus abdominal instead of local venous congestion is induced. The constriction is permanent and the model is suitable for both acute and chronic follow-ups. Importantly, this study confirmed that conventional echocardiographic parameters and cardiac hemodynamics were largely unaffected after surgery, thereby excluding the effects of forward failure (i. e., decreased cardiac output)in our rat model^16^. This gives the unique possibility to study the pathophysiology of selective backward failure, in contrast to the model of Fujimoto *et al*.^17^. In this previous study, right-sided heart failure was induced in rats by pulmonary artery banding and after four weeks of follow-up, hepatic function was investigated. The novelty of this study is emphasized by the fact that in the current model right-sided cardiac function and cardiac preload were not compromised and both cardiac, renal and hepatic function were examined in depth after twenty-one weeks of follow-up.

Moreover, abdominal venous congestion induces a systemic inflammation, as evidenced by the significantly increased plasma CRP levels in IVCc rats. Inflammation is an important connector in the cardiorenal syndrome^18^ and recently, it was shown in patients that peripheral congestion causes release of inflammatory mediators^19^. In the current rat model, inflammation could be an important contributor to the observed worsening in kidney and liver function, as described in the next sections.

### Abdominal venous congestion alters renal morphology

In IVC-constricted rats, it was demonstrated that adverse alterations occur in the kidneys, since both the width of Bowman’s space and the glomerular surface area were significantly increased after 21 weeks, similar to the 12-week follow-up study in a previous study by Cops *et al*.^15^. and as reported by Dong *et al*.^20^. Glomerular surface area correlated significantly with abdominal venous pressure, suggesting a causal relationship. In our observations, glomerular density was comparable in both SHAM and IVCc rats. As already explained in the study of Cops *et al*., these results fit with “retrogradely transduced glomerular hypertension without major impact on the glomerular filtration rate (GFR)^15^. Theoretically, renal congestion leads to tubular compression and an augmented luminal pressure and in this way the transglomerular pressure gradient and GFR are lowered^3,8,21^. in an attempt to increase the single nephron GFR, afferent vasodilation and efferent vasoconstriction might have been initiated^22^, resulting in enlarged glomeruli and intraglomerular hypertension”^15^. Hypothetically, hyperfiltration may develop as a response to intraglomerular hypertension thereby mediating progressive kidney damage, as already demonstrated in various disease settings^23^. In addition, plasma and urinary urea excretion and urea clearance were unchanged in IVCc rats, suggesting a preserved renal blood flow. However, these mechanisms are maybe too simplistic and alterations in lymph flow and intrarenal hemodynamic changes may also play an important role in this aspect^24^.

It was found that IVCc rats demonstrated increased plasma creatinine levels twenty-one weeks after surgery. In heart failure patients, an elevated central venous pressure provokes renal congestion^8^ and consequently renal dysfunction, since blood flow through the kidneys is reduced more by an increase in venous pressure than by an equivalent decrease in arterial pressure^25^. The discrepancy between the rise in plasma creatinine and the unchanged levels of cystatin C might be explained by a higher creatinine production or less tubular secretion of creatinine. The 24 h creatinine excretion found in the urine collections of IVCc rats was not different from SHAM rats. Calculated renal creatinine clearance did not differ significantly between both experimental groups, due to large variations in individual urinary creatinine excretion levels. Next to an upregulated inflammatory status, activation of the RAAS system and oxidative stress are suggested to be important cardiorenal connectors^26^. However, no evidence of RAAS hyperactivation was observed since plasma aldosterone and protein expression levels of ATIIT1R did not differ between both groups. Protein expression levels of NOX2, a membrane complex responsible for reactive oxygen species production, also did not differ between both groups, suggesting a preserved redox balance. In our model, glomerular density as well as cardiac function were preserved. This implies renal glomerular adaptation masking relevant effects of the abdominal venous congestion on creatinine clearance after 21 weeks. In our previous study^15^, we demonstrated significantly increased plasma cystatin C levels and urinary albumin levels in IVCc rats, however significance of both parameters disappeared in the current study. This implies a possible adaptation mechanism of the kidney whereby the kidney is able to cope provisionally with the renal venous congestion thanks to the aforementioned glomerulomegaly.

In this study, there was no evidence of increased renal fibrosis or tubular damage, indicating isolated hemodynamically mediated alterations of function. The lack of pronounced renal implications may be explained by the fact that nephrons are adapting to the increased renal interstitial pressure and have not been destroyed yet or by the fact that the increased abdominal venous pressure possibly did not reach a value sufficient to increase intrarenal pressure, thereby causing renal hypoperfusion and tubular damage. Moreover, abdominal venous congestion was induced in an otherwise healthy rat, in contrast to patients which already have developed heart and/or kidney failure before displaying signs and symptoms of congestion, and it is notoriously difficult to develop a rodent model of albuminuria or renal failure. To summarize, kidney function seems to adapt after 21 weeks of abdominal venous congestion. However, by prolonging the observational period, renal glomerular and tubular damage and fibrosis may occur and the already raised plasma creatinine levels could represent the initial sign of renal deterioration.

### Abdominal venous congestion induces hepatic fibrosis

Hepatic dysfunction is a frequent complication of right-sided heart failure in patients since an elevated CVP causes passive hepatic congestion, which is referred to as congestive hepatopathy^17,27^, resulting in increased levels of bilirubin^28^. We reported a significantly greater plasma bilirubin level, liver weight/tibia length weight ratio and spleen weight/tibia length ratio in IVC-constricted rats after 21 weeks of follow-up, indicating clinically meaningful hepatic congestion resulting from the constriction, probably due to the fact that the liver is the first organ affected by the increased abdominal venous pressure. This may have clinical implications, as an elevated serum bilirubin is a risk factor for premature death in patients with pulmonary arterial hypertension and right-sided heart failure^29^. An increased spleen weight/tibia length ratio is defined in literature as an indicator of portal hypertension and reflects splanchnic system involvement^16,30^. Due to abdominal venous congestion and portal hypertension, hepatic hydrostatic pressure increases and results in edema and hemorrhage, thereby compromising oxygenation and eventually inducing hepatocellular necrosis^17,27^. The pathological appearance of a liver affected by venous congestion is speckled and known as a ‘nutmeg liver’^31^, which was also observed in this study, suggesting development of congestive hepatopathy^17,27^.

The Masson trichrome staining indicated a marked increase in hepatic collagen deposition with a periportal to centrilobular distribution pattern and development of fibrous septa. When liver damage is persistent and progressive, hepatic regeneration is halted and hepatic fibrosis develops. In time, progressive fibrosis results in cirrhosis. Congestion induces hepatocellular necrosis and results in increased transforming growth factor β (TGF-β) production by Kupffer cells, which activates hepatic stellate cells (HSCs)^32^. Activated HSCs transform to α-SMA-positive myofibroblasts, responsible for collagen I and III synthesis and thus promoting hepatic fibrosis^17,33^. Activation of HSCs is observed in this study based on a significantly greater hepatic α-SMA protein expression in IVCc rats after 21 weeks. In addition, the increase in α-SMA protein expression correlates significantly with the abdominal venous pressure. The augmented abdominal venous pressure leads to hepatic sinusoidal congestion which clinically contributes to development of fibrosis in congestive hepatopathy, possibly through the mechanism of sinusoidal thrombosis, according to Simonetto *et al*.^16^. but contradicted by Fujimoto *et al*.^17^. The increased collagen deposition creates a physical impairment to the bidirectional flow of plasma between the hepatic sinusoidal lumen and hepatocytes, ultimately altering hepatic function and liver congestion^34^.

In acute decompensated heart failure (ADHF) patients, an increased venous pressure is reflected by an augmented liver stiffness, ultimately also contributing to hepatic fibrosis. In non-alcoholic fatty liver disease patients, the fibrosis-4 index is known as a marker of liver stiffness^35^. Recently, it was demonstrated in heart failure patients that the fibrosis-4 index was associated with hyaluronic acid, type IV collagen 7S, right and left heart volume overload, brain natriuretic peptide (BNP) and higher all-cause mortality^36^. Hence, an increased FIB4 index is also a marker of liver stiffness in ADHF patients and is indicative of hepatic fibrosis due to underlying congestion^36^. Besides an increased liver stiffness, abdominal venous congestion causes remodeling of the extracellular matrix (ECM), during which collagen fragments are deposited into the general circulation. 7S domain of collagen type IV (P4NP 7S), expressed in the basement membrane of the hepatic ECM, is such a released fragment and has been shown to be a marker of hepatic fibrogenesis^37^. In heart failure patients, P4NP 7S correlated with BNP, right-sided cardiac pressure, pulmonary capillary wedge pressure and gamma-glutamyltransferase, indicating an accelerated production of hepatic collagen type IV and ECM remodeling. Since cardiac index was not correlated with P4NP 7S, it was concluded that the accelerated turnover should be attributed to presence of congestion. Third, right ventricular dysfunction and concomitant systemic congestion can also result from pulmonary hypertension. Yoshihisa *et al*. demonstrated that serum P4NP 7S correlated with right-sided volume overload and an increased central venous pressure and that PHNP 7S was associated with higher mortality in pulmonary hypertension patients^38^. Since increased P4NP 7S levels reflect ECM remodeling resulting from congestion-induced organ injury, this particular collagen fragment can be applied to investigate hepatic fibrosis^37,38^.

Based on the aforementioned arguments, the importance of backward failure caused by right-sided heart failure or hepatic congestion in the pathophysiology of congestive hepatopathy, is highlighted. To summarize, abdominal venous congestion leads in the current rat model to hepatic deterioration over time. In the same model, kidney function adapts after 21 weeks, suggesting that the liver is more susceptible or vulnerable to abdominal congestion. The clinical implications for patients are that after normalization of an increased abdominal venous pressure, kidney function and morphology is restored, in contrast to a chronically altered liver function and morphology.

### Limitations

This study had a maximal follow-up of 21 weeks, so only conclusions on the short to middle-term effects of abdominal venous congestion can be deferred. In the future, the model may be investigated for a longer period of time to investigate if renal glomerular and tubular damage and fibrosis occurs. Second, renal blood flow was not assessed. Third, GFR was only assessed by creatinine clearance and evaluation by inulin clearance is lacking. Fourth, markers reflecting sympathetic nervous activity should be provided, since this is an important cardiorenal connector. Fifth, assessment of cardiac function was focused on the left-sided heart. However, parameters of right-sided cardiac function may also deviate as a result of the constriction and should be investigated in the future. Finally, a comprehensive assessment of liver function is lacking.

## Conclusion

This study demonstrated that abdominal venous congestion induces glomerulomegaly, suggesting retrogradely transduced glomerular hypertension with hyperfiltration and without major impact on the glomerular filtration rate. In addition, liver fibrosis was observed in this model. The observed kidney and liver dysfunction may be attributed to an upregulated inflammatory status. Importantly, cardiac function remained comparable between both groups, excluding forward failure as the reason for the observations. Thus a rat model is now available to study the influence of abdominal venous congestion *per se* in congestion-related diseases, independent of cardiac output or underlying kidney function.

## Material and Methods

### Animals and housing

This study conforms to the EU Directive 2010/63/EU for animal experiments and was approved by the Ethical Committee for Animal Experiments of Hasselt University, Belgium (protocol number: 201553A1). Rats had *ad libitum* access to food and water and were maintained in a temperature (22 °C) and light (12:12 h cycle) controlled animal facility^15^.

### Study design

Forty rats (male Sprague-Dawley, 135 ± 15 g, Charles River, France) were divided into 2 groups: twelve sham-operated rats (SHAM group) were compared to eleven rats subjected to inferior vena cava (IVC) constriction (IVCc group) and both groups were studied for 21 weeks. The perioperative mortality rate in the IVCc group was 61% (17/28) compared to 0% in the SHAM group (0/12). Abdominal venous congestion was induced by increasing the abdominal venous pressure through surgical constriction of the thoracic IVC^15^.

### Experimental protocol

Surgical constriction of the IVC was applied as described before by Cops *et al*.^15^. Briefly, rats were intubated and anesthetized (1.5% isoflurane volume supplemented with oxygen) and a right anterolateral thoracotomy was performed. The thoracic IVC was visualized to apply a permanent constriction by tying a surgical wire (6–0 prolene, VMD, Belgium) around the IVC and a 20 gauge (0.812 mm) needle. Afterwards, the 20 G needle was removed and the wound was closed. The same surgical procedure was applied in sham-operated rats except for application of the constriction. Meloxicam (1 mg/kg, Boehringer, Germany) was administered subcutaneously pre-operatively and was continued postoperatively twice a day for three consecutive days, while antibiotics (10 mg/kg/day, Baytril, Bayer, Belgium) were administered for 5 consecutive days postoperatively via the drinking water. At 21 weeks after surgery, rats were weighed, 24 h urine samples were collected using standard rodent metabolic cages (Technilab-BMI, the Netherlands), blood samples were obtained from the tail artery and echocardiography was performed, both under isoflurane anesthesia (1.5–2% volume supplemented with oxygen). After performing invasive hemodynamic measurements, rats were sacrificed with an overdose of pentobarbital (200 mg/kg, i.p.). and heart, kidneys and liver were excised for histological and molecular examination. Before embedding in paraffin, tissue sections were fixed overnight in 4% paraformaldehyde and conserved in 70% ethanol. Residual tissues were crushed to a fine powder, snap frozen in liquid nitrogen and stored at −80 °C^15^.

### Blood and urine biochemical analysis

As described in Cops *et al*., blood samples were centrifuged (2000 rpm, 10 min) and plasma was preserved (−20 °C) for later analysis. Plasma samples were analyzed for bilirubin, creatinine, urea, cystatin C, aldosterone and C-reactive protein (CRP) using an automated analyzer (Cobas 8000 ISE module and Cobas 8000 c702 and c502 module, Roche Diagnostics, Germany)^15,39–41^. Urine samples were centrifuged (1500 rpm, 5 min) and preserved (−20 °C) for later analysis. Urine samples were analyzed for creatinine, urea and albumin using an automated analyzer (Cobas 8000 ISE module and Cobas 8000 c702 and c502 module, Roche Diagnostics, Germany). Urinary kidney injury molecule 1 (KIM-1) concentrations were determined using the rat TIM-1/KIM-1/HAVCR DuoSet ELISA kit (DY3689, R&D Systems, USA) according to the manufacturer’s instructions and all measurements were performed in duplicate^15^. Creatinine clearance (ml/min/kg) was calculated as follows = [(urinary creatinine (mg/dl) × urinary volume (ml/24 h))/(plasma creatinine (mg/dl) × 1440 min) * 1000]/body weight^15,42^. Likewise, urea clearance was calculated.

### Echocardiography measurements

Left ventricular function was the primary outcome to assess cardiac function in response to the constriction. Left ventricular echocardiography was performed at baseline and 21 weeks after surgery using the GE VIVID *i* ultrasound machine and a 10S transducer (GE Vingmed Ultrasound, version 7.0.1, Norway), under isoflurane anesthesia in spontaneously breathing rats (1.5–2% volume supplemented with oxygen), as described previously^15^. In B-mode at a temporal resolution of ≈200 frames per second, a standard parasternal long-axis image and a short-axis image at midventricular level were acquired. The following parameters were obtained from the parasternal short-axis view: left ventricular end-diastolic diameter (LVEDD), LV end-systolic diameter (LVESD), posterior and anterior wall thicknesses (PWT, AWT). Left ventricular end-diastolic volumes (EDV) and LV end-systolic volumes (ESV) were calculated by π * D_M_^2^ * B/6. D_M_ indicates the systolic/diastolic diameter of the ventricle on midventricular short-axis view and B is the LV length on the parasternal long-axis image. Heart rate (HR) was determined by defining end systole and end diastole as the minimum and maximum LV short-axis area, respectively. Stroke volume (SV) was calculated as EDV – ESV. Cardiac output (CO) was calculated as SV * HR (EchoPAC workstation, GE Vingmed Ultrasound, version 7.0.1, Norway)^15^.

### Venous and arterial hemodynamic measurements

At the end of the experimental period of 21 weeks, rats were subjected to invasive venous and arterial hemodynamic measurements. After induction of anesthesia (1.5–2% isoflurane volume supplemented by oxygen), a calibrated 2F micro tip high-fidelity pressure catheter (Millar Instruments, AD Instruments, Germany), was inserted into the right jugular vein to obtain jugular venous pressure. Next, the left femoral vein was cannulated and the catheter was advanced into the abdominal IVC to obtain abdominal venous pressure. Third, LV pressure (LVP) and left ventricular end-diastolic pressure (LVED) were recorded by inserting the catheter in the right carotid artery and advancing the catheter into the left ventricle. The peak time derivatives (dP/dt_max_ and dP/dt_min_) and the time constant of LV pressure decay during the isovolumic relaxation period (tau) were calculated using LabChart v7.3.7 software (Millar Instruments, AD Instruments, Germany). Afterwards, rats were sacrificed with an overdose of pentobarbital (200 mg/kg, i.p.)^15^.

### Fibrosis measurement

Five µm thickness sections of liver, kidney and heart tissue, subjected to the Masson trichrome staining method, were scanned using the Mirax Desk (Carl Zeiss MicroImaging, Germany). Fibrosis was assessed at 20X magnification in four randomly chosen sections in each organ per rat, by outlining the area of collagen deposition and excluding blood vessels, as described previously^15,43^. Quantification of percentage collagen was performed by calculating the ratio of the area of collagen deposition to the global area using an automated image analysis program (AxioVision 4.6, Carl Zeiss MicroImaging, Germany)^15^.

### Kidney morphology

Kidney morphology was assessed in kidney sections subjected to the Masson trichrome staining method, as described previously^15^. Glomerular surface area was measured in 10 randomly chosen glomeruli per rat and width of Bowman’s space was measured 5 times per Bowman’s space in 10 randomly chosen glomeruli per rat. Glomerular density was calculated by counting well-preserved glomeruli in 5 randomly selected fields with a surface area of 3.14 mm² in renal sections of each rat using an analysis program (Pannoramic Viewer, 3DHISTECH, Hungary)^15,44–46^.

### Western blot

Protein concentrations of liver and kidney samples were determined using the BCA protein assay kit (Thermo Fisher, Belgium). Samples containing the same amount of proteins were separated on a 12% SDS-page gel with a mini protean 3 electrophoresis system (Bio-rad Laboratories, Belgium), then transferred to a polyvinylidene fluoride (PVDF) membrane and blocked two hours with 5% milk in Tris-buffered solution containing 0.1% Tween-20 (TBS-T) or 5% bovine serum albumin (BSA) in TBS-T, depending on the primary antibody. The membrane was incubated overnight at 4 °C in the presence of an alpha-smooth muscle actin antibody (α-SMA, 1/2000, ab5694, Abcam, UK), anti-angiotensin II type I receptor antibody (ATIIT1R, 1/2000, ab18801, Abcam, UK) or NAPDH oxidase 2 antibody (NOX2, 1/2000, ab31092, Abcam, UK). Secondary swine anti-rabbit horseradish peroxidase-conjugated antibody (P0217, DAKO, Belgium) at a dilution of 1/2500 was used. Both primary and secondary antibodies for α-SMA and NOX2 were diluted in 5% milk-TBS-T, while primary and secondary antibodies for ATIIT1R were diluted in 5% BSA-TBS-T. α-SMA, ATIIT1R and NOX2 were visualized using the chemiluminescence (ECL) technique using the Pierce ECL Plus Western Blotting Substrate Kit (Thermo Fisher, Belgium) and quantified using Image Quant TL software v8.1 (GE Healthcare Europe, Belgium). Data were normalized to GAPDH protein levels (1/2000, MA5–15738-HRP, Thermo Fisher, Belgium) or to β-actin protein levels (1/2500, sc-4778, Santa Cruz, USA). The original full-length western blots of ATIIT1R, NOX2 and α-SMA are shown in Supplementary Figs 2–4.

### Immunohistochemistry

Five-µm-thick hepatic tissue sections were deparaffinized in xylene and rehydrated serially with alcohol and water, followed by microwave antigen retrieval for 20 min at 98 °C in 10 mM sodium citrate buffer (0.05% tween 20, pH 6.0). Endogenous peroxidase was blocked with fresh 0.3% hydrogen peroxide in phosphate buffered saline (PBS) for ten minutes at room temperature. Sections were blocked 2 h with protein block (DAKO, Belgium) in PBS containing 0.5% triton and were incubated overnight at 4 °C in the presence of a specific alpha-smooth muscle actin antibody (α-SMA, 1/200, ab5694, Abcam, UK). After being washed three times in PBS, sections were incubated with biotinylated secondary swine anti-rabbit antibody (E0431, DAKO, Belgium) for 1 h and with streptavidine-HRP (1/800, P0397, DAKO, Belgium) for 30 min. α-SMA was visualized using DAB (K3468, DAKO, Belgium) and sections were counterstained with hematoxylin. Sections were observed at 20X magnification Mirax Desk and Mirax viewer, Carl Zeiss MicroImaging, Germany).

### Statistical analysis

Data are expressed as median [25^th^ percentile; 75^th^ percentile]. Normality was tested using the Shapiro-Wilk normality test. Data were analyzed using an unpaired t-test or a Mann-Whitney test as appropriate. Relations were examined by Pearson’s r or Spearman’s ρ as appropriate. A 2-tailed value of p < 0.05 was considered statistically significant^15^. Statistical analysis was performed using GraphPad Prism (GraphPad Prism Software 7.04, USA). The observed statistical power of inter-group comparisons was calculated by use of G*Power 3.1.9.2 (Universität Düsseldorf, Germany). An observed power ≥0.80 was considered sufficient.

## Electronic supplementary material

Supplemental figures

## References

[1] Mullens W (2009). Importance of venous congestion for worsening of renal function in advanced decompensated heart failure. J Am Coll Cardiol.

[2] Dupont M, Mullens W, Tang WH (2011). Impact of systemic venous congestion in heart failure. Curr Heart Fail Rep.

[3] Nijst P, Mullens W (2014). The acute cardiorenal syndrome: burden and mechanisms of disease. Curr Heart Fail Rep.

[4] Marik PE, Baram M, Vahid B (2008). Does central venous pressure predict fluid responsiveness? A systematic review of the literature and the tale of seven mares. Chest.

[5] Harjola VP (2017). Organ dysfunction, injury and failure in acute heart failure: from pathophysiology to diagnosis and management. A review on behalf of the Acute Heart Failure Committee of the Heart Failure Association (HFA) of the European Society of Cardiology (ESC). European journal of heart failure.

[6] Mullens W (2008). Prompt reduction in intra-abdominal pressure following large-volume mechanical fluid removal improves renal insufficiency in refractory decompensated heart failure. J Card Fail.

[7] Mullens W (2008). Elevated intra-abdominal pressure in acute decompensated heart failure: a potential contributor to worsening renal function?. J Am Coll Cardiol.

[8] Afsar B (2016). Focus on renal congestion in heart failure. Clinical kidney journal.

[9] Gnanaraj JF, von Haehling S, Anker SD, Raj DS, Radhakrishnan J (2013). The relevance of congestion in the cardio-renal syndrome. Kidney Int.

[10] Chen KP (2016). Peripheral Edema, Central Venous Pressure, and Risk of AKI in Critical Illness. Clin J Am Soc Nephrol.

[11] Armaly Z, Abassi Z (2014). Deleterious Effects of Increased Intra-Abdominal Pressure on Kidney Function. Advances in Nephrology ID.

[12] Mohmand H, Goldfarb S (2011). Renal dysfunction associated with intra-abdominal hypertension and the abdominal compartment syndrome. J Am Soc Nephrol.

[13] Adams KF (2005). Characteristics and outcomes of patients hospitalized for heart failure in the United States: rationale, design, and preliminary observations from the first 100,000 cases in the Acute Decompensated Heart Failure National Registry (ADHERE). American heart journal.

[14] Damman K (2010). Congestion in chronic systolic heart failure is related to renal dysfunction and increased mortality. European journal of heart failure.

[15] Cops J (2018). Selective abdominal venous congestion to investigate cardiorenal interactions in a rat model. PloS one.

[16] Simonetto DA (2015). Chronic passive venous congestion drives hepatic fibrogenesis via sinusoidal thrombosis and mechanical forces. Hepatology (Baltimore, Md.).

[17] Fujimoto Y (2016). Low Cardiac Output Leads Hepatic Fibrosis in Right Heart Failure Model Rats. PloS one.

[18] Kingma John, Simard Denys, Rouleau Jacques, Drolet Benoit, Simard Chantale (2017). The Physiopathology of Cardiorenal Syndrome: A Review of the Potential Contributions of Inflammation. Journal of Cardiovascular Development and Disease.

[19] Colombo PC (2014). Peripheral venous congestion causes inflammation, neurohormonal, and endothelial cell activation. European heart journal.

[20] Dong Z (2015). Myocardial infarction accelerates glomerular injury and microalbuminuria in diabetic rats via local hemodynamics and immunity. International journal of cardiology.

[21] Ross EA (2012). Congestive renal failure: the pathophysiology and treatment of renal venous hypertension. Journal of cardiac failure.

[22] Sraer JD, Kanfer A, Rondeau E, Lacave R (1989). Role of the renin-angiotensin system in the regulation of glomerular filtration. Journal of cardiovascular pharmacology.

[23] Helal I, Fick-Brosnahan GM, Reed-Gitomer B, Schrier RW (2012). Glomerular hyperfiltration: definitions, mechanisms and clinical implications. Nature reviews. Nephrology.

[24] Verbrugge FH (2013). Abdominal contributions to cardiorenal dysfunction in congestive heart failure. J Am Coll Cardiol.

[25] Ganda A (2010). Venous congestion and endothelial cell activation in acute decompensated heart failure. Current heart failure reports.

[26] Bongartz LG, Cramer MJ, Doevendans PA, Joles JA, Braam B (2005). The severe cardiorenal syndrome: ‘Guyton revisited’. European heart journal.

[27] Shah SC, DA S (2015). “Cardiac Hepatopathy”: A Review of Liver Dysfunction in Heart Failure. Liver Res Open J.

[28] Alvarez AM, Mukherjee D (2011). Liver abnormalities in cardiac diseases and heart failure. The International journal of angiology: official publication of the International College of Angiology, Inc.

[29] Takeda Y (2010). Bilirubin as a prognostic marker in patients with pulmonary arterial hypertension. BMC pulmonary medicine.

[30] Huebert RC (2011). Aquaporin-1 promotes angiogenesis, fibrosis, and portal hypertension through mechanisms dependent on osmotically sensitive microRNAs. The American journal of pathology.

[31] Sherlock S (1951). The liver in heart failure; relation of anatomical, functional, and circulatory changes. British heart journal.

[32] Dooley S, ten Dijke P (2012). TGF-beta in progression of liver disease. Cell and tissue research.

[33] Seki E, Brenner DA (2015). Recent advancement of molecular mechanisms of liver fibrosis. Journal of hepato-biliary-pancreatic sciences.

[34] Hernandez-Gea V, Friedman SL (2011). Pathogenesis of liver fibrosis. Annual review of pathology.

[35] Shah AG (2009). Comparison of noninvasive markers of fibrosis in patients with nonalcoholic fatty liver disease. Clinical gastroenterology and hepatology: the official clinical practice journal of the American Gastroenterological Association.

[36] Sato Y (2017). Liver stiffness assessed by Fibrosis-4 index predicts mortality in patients with heart failure. Open heart.

[37] Nagao K (2017). Liver fibrogenesis marker, 7S domain of collagen type IV in patients with acutely decompensated heart failure: Correlates, prognostic value and time course. Int J Cardiol.

[38] Yoshihisa A (2018). Liver fibrosis marker, 7S domain of collagen type IV, in patients with pre-capillary pulmonary hypertension. Int J Cardiol.

[39] Vaidya VS (2010). Kidney injury molecule-1 outperforms traditional biomarkers of kidney injury in preclinical biomarker qualification studies. Nature biotechnology.

[40] Zhao Z (2016). Sulforaphane Attenuates Contrast-Induced Nephropathy in Rats via Nrf2/HO-1 Pathway. Oxidative medicine and cellular longevity.

[41] Erdem A (2000). The protective effect of taurine against gentamicin-induced acute tubular necrosis in rats. Nephrology, dialysis, transplantation: official publication of the European Dialysis and Transplant Association - European Renal Association.

[42] Rafiq K (2012). Renal sympathetic denervation suppresses de novo podocyte injury and albuminuria in rats with aortic regurgitation. Circulation.

[43] Ichinose F (2004). Pressure overload-induced LV hypertrophy and dysfunction in mice are exacerbated by congenital NOS3 deficiency. American journal of physiology. Heart and circulatory physiology.

[44] Kanzaki G (2013). Distribution of glomerular density in different cortical zones of the human kidney. Pathology international.

[45] de Vries WB (2010). Neonatal dexamethasone treatment in the rat leads to kidney damage in adulthood. Pediatric research.

[46] Barbuto N, Almeida JR, Pereira LM, Mandarim-de-Lacerda CA (2004). Renal cortex remodeling in nitric oxide deficient rats treated with enalapril. Journal of cellular and molecular medicine.

