# Supplementary material for: Selective abdominal venous congestion induces adverse renal and hepatic morphological and functional alterations despite a preserved cardiac function
Source: Sci Rep. 2018 Dec 10;8:17757. doi: 10.1038/s41598-018-36189-3 (PMC6288122; doi:10.1038/s41598-018-36189-3)
Supplement: Supplementary file 1 — Supplemental figures [file 41598_2018_36189_MOESM1_ESM.pdf]

Selective abdominal venous congestion induces adverse renal and hepatic morphological and functional alterations despite a preserved cardiac function

Jirka Cops[1,2*], Wilfried Mullens[1,3], Frederik H. Verbrugge[3], Quirine Swennen[1], Bart De Moor[1,4], Carmen Reynders[5], Joris Penders[1,5], Ruth Achten[1,6], Ann Driessen[7], Amélie Dendooven[7], Jean-Michel Rigo[1], Dominique Hansen[1,8,9]

[1]BIOMED – Biomedical Research Institute, Faculty of Medicine and Life Sciences, Hasselt University, Diepenbeek, Belgium

[2]Doctoral school for Medicine and Life Sciences, Hasselt University, Diepenbeek, Belgium

[3]Department of Cardiology, Ziekenhuis Oost-limburg, Genk, Belgium

[4]Department of Nephrology, Jessa Ziekenhuis, Hasselt, Belgium

[5]Clinical laboratory, Ziekenhuis Oost-Limburg, Genk, Belgium

[6]Department of Pathology, Jessa Ziekenhuis, Hasselt, Belgium

[7]Department of Pathology, Universitair Ziekenhuis Antwerpen, University of Antwerp, Edegem, Belgium

[8]REVAL, – Rehabilitation Research Center, Faculty of Rehabilitation Sciences, Hasselt University, Diepenbeek, Belgium

[9]Heart Centre Hasselt, Jessa Hospital, Hasselt, Belgium

*Corresponding author at: Hasselt University, BIOMED, Martelarenlaan 42, BE-3500 Hasselt, Belgium

E-mail address: jirka.cops@uhasselt.be / +32(0)11 26 92 55

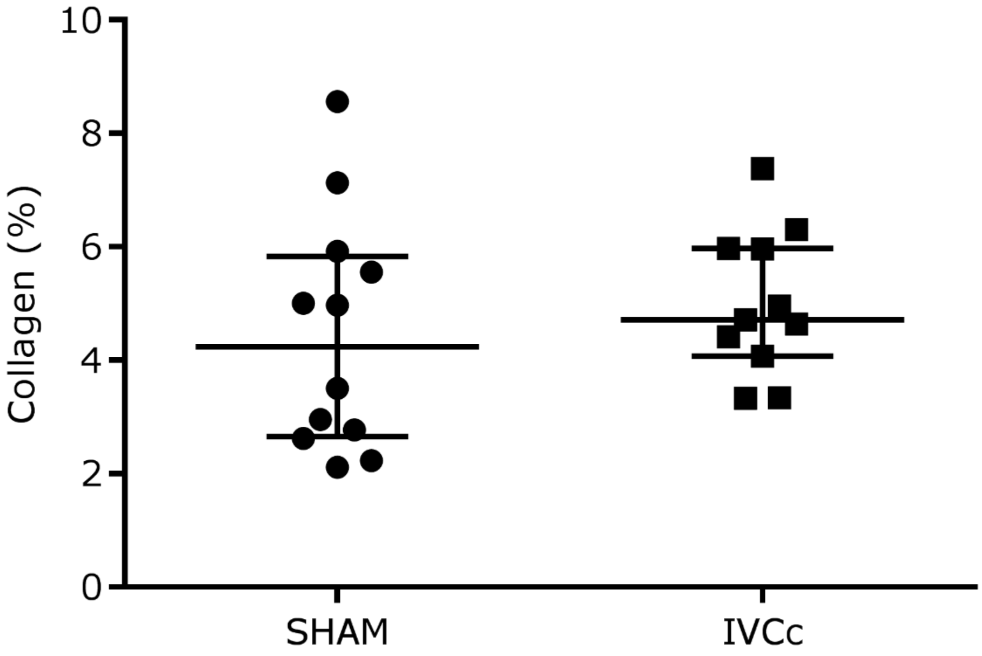

Fig S1. Cardiac collagen deposition is not affected by abdominal venous congestion. Quantification of total collagen from transverse heart sections, stained with a Masson trichrome staining, of sham-operated (SHAM, n=12) and IVC-constricted rats (IVCc, n=11), twenty-one weeks after surgery. Data were analyzed using an unpaired t-test, based on the Shapiro-Wilk normality test. Data are shown as median, 25th percentile, 75th percentile, minimum and maximum. IVC = inferior vena cava, IVCc = IVC-constricted rats.

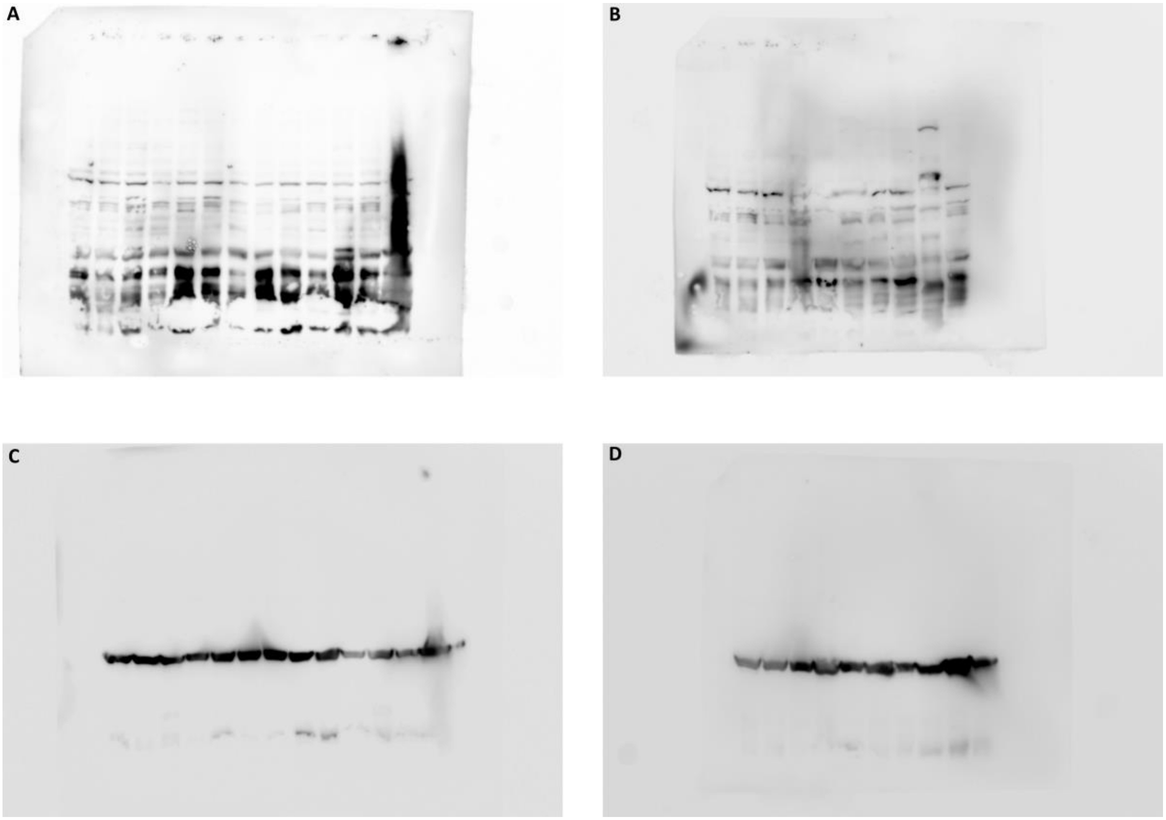

Fig S2. Full-length western blot for renal ATIIT1R and β-actin. (A, B) representative western blot for hepatic ATIIT1R and (C, D) representative western blot for hepatic β-actin of sham-operated (SHAM, n=12) and IVC-constricted rats (IVCc, n=11), twenty-one weeks after surgery. Samples were derived from the same animal experiment and blots were processed in parallel. Due to lack of space, samples were dived over two gels. After detection of ATIIT1R, blots were striped to detect β-actin as a loading control. These blots are the original and unprocessed blots. IVC = inferior vena cava, IVCc = IVC-constricted rats, ATIIT1R = angiotensin II type I receptor.

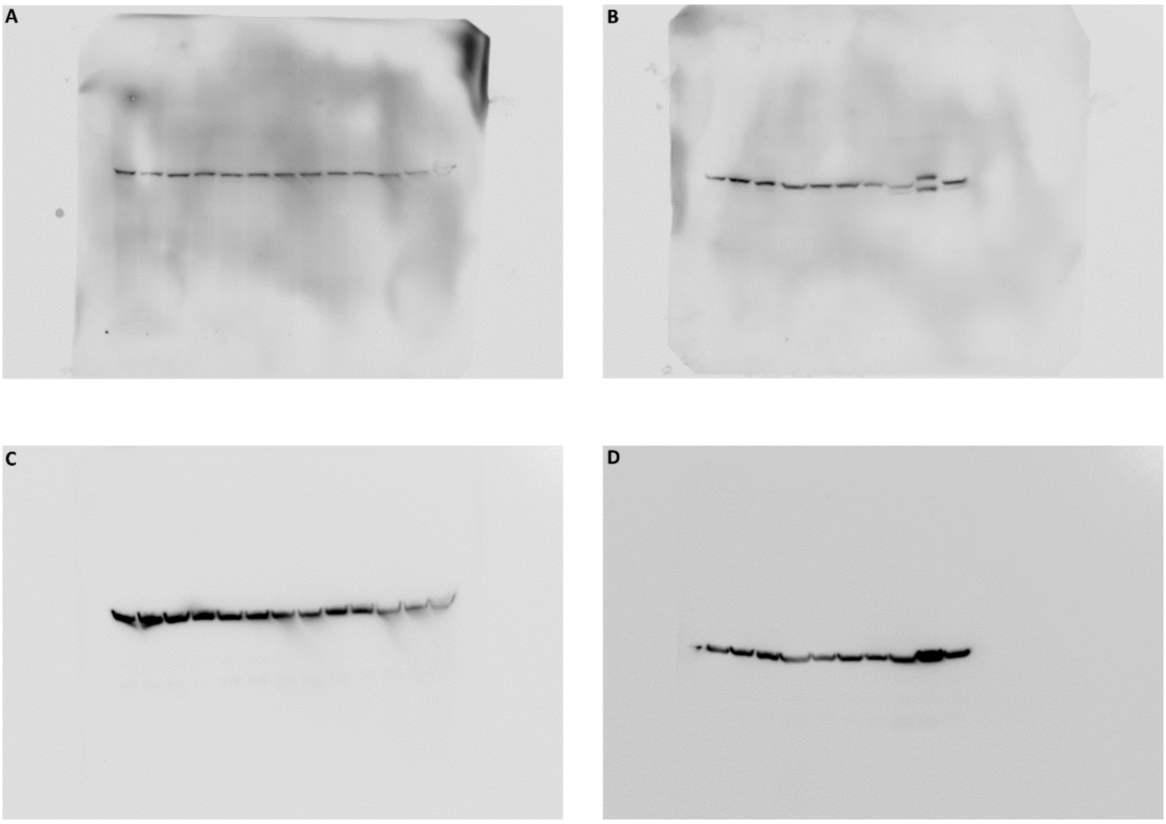

Fig S3. Full-length western blot for renal NOX2 and β-actin. (A, B) representative western blot for renal NOX2 and (C, D) representative western blot for renal β-actin of sham-operated (SHAM, n=12) and IVC-constricted rats (IVCc, n=11), twenty-one weeks after surgery. Samples were derived from the same animal experiment and blots were processed in parallel. Due to lack of space, samples were dived over two gels. After detection of NOX2, blots were striped to detect β-actin as a loading control. These blots are the original and unprocessed blots. IVC = inferior vena cava, IVCc = IVC-constricted rats, NOX2 = NAPDH oxidase 2.

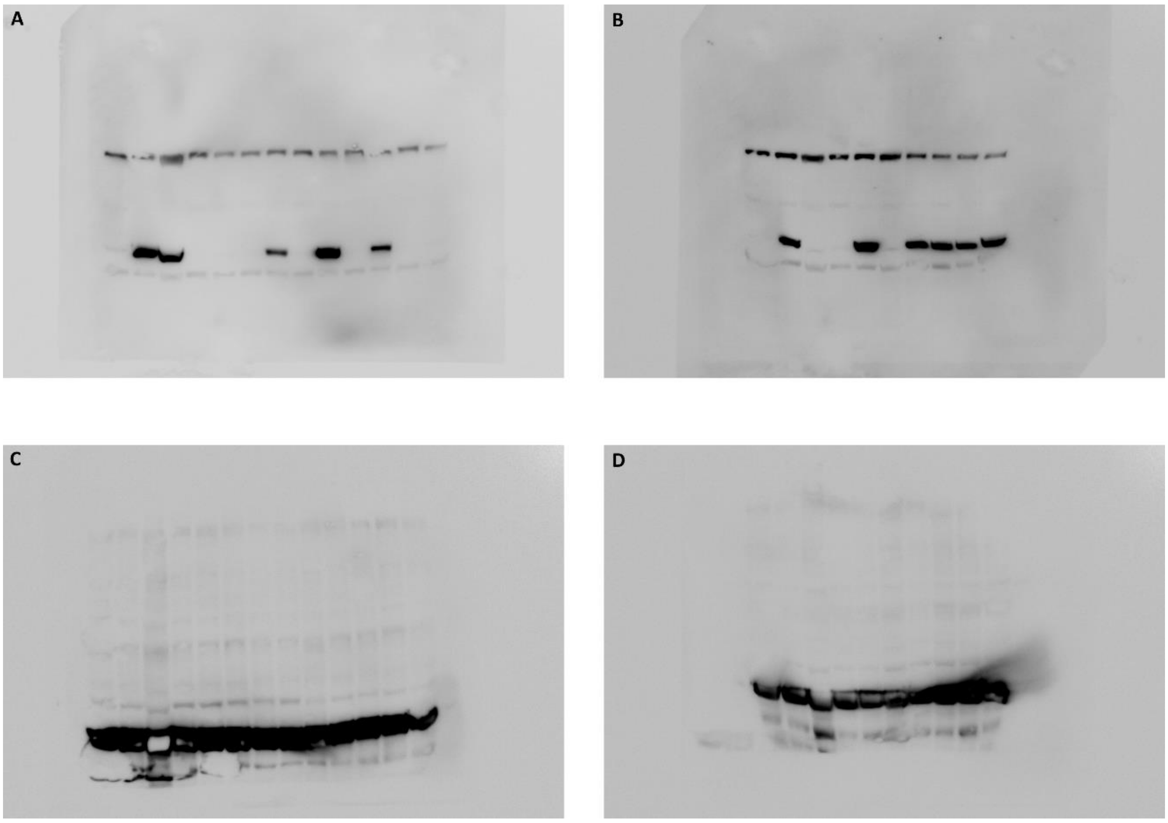

Fig S4. Full-length western blot for hepatic α-SMA and GAPDH. (A, B) representative western blot for hepatic α-SMA and (C, D) representative western blot for hepatic GAPDH of sham-operated (SHAM, n=12) and IVC-constricted rats (IVCc, n=11), twenty-one weeks after surgery. Samples were derived from the same animal experiment and blots were processed in parallel. Due to lack of space, samples were dived over two gels. After detection of α-SMA, blots were striped to detect GAPDH as a loading control. These blots are the original and unprocessed blots. IVC = inferior vena cava, IVCc = IVC-constricted rats, α-SMA = alpha-smooth muscle actin.
